# Long-term outcomes in major trauma patients and correlations with the acute phase

**DOI:** 10.1186/s13017-020-0289-3

**Published:** 2020-01-13

**Authors:** Costanza Martino, Emanuele Russo, Domenico Pietro Santonastaso, Emiliano Gamberini, Silvia Bertoni, Emanuele Padovani, Luigino Tosatto, Luca Ansaloni, Vanni Agnoletti

**Affiliations:** 1 Anesthesia and Intensive Care Unit, AUSL Romagna, M.Bufalini Hospital, Viale Ghirotti 286 - 47521, Cesena, Italy; 2 General and Emergency Surgery Unit, AUSL Romagna, M.Bufalini Hospital, Cesena, Italy; 3 University of Bologna, Bologna, Italy; 4 Department of Management—DiSA, University of Bologna, Bologna, Italy; 5 Department of Neurosurgery, AUSL Romagna, M.Bufalini Hospital, Cesena, Italy

**Keywords:** Trauma, Trauma care, Outcomes, Long-term outcome, Disability, Quality of life

## Abstract

**Background:**

Major trauma patients experience a 20% mortality rate overall, and many survivors remain permanently disabled.

In order to monitor the quality of trauma care in the Trauma System, outcomes assessment is essential. Quality indicators on outcome can be expressed as quality of life, functional outcome, and others.

The trauma follow-up system was created within the Romagna Trauma System (Italy) in order to monitor the trauma network and assess its long-term outcomes.

The aim of this paper is firstly to evaluate the existence of correlations between epidemiological data, severity of injury, and clinical assessment characterizing the acute phase and the long-term outcomes in trauma patients and secondly, to explore the association between outcome variables have been modified.

**Methods:**

We conducted a cross-sectional study over a 10-year period, including patients with severe trauma who survived and were discharged from the intensive care unit. The outcome measures were assessed with the use of the Extended Glasgow Outcome Scale and the Euro Quality of Life scale 5 dimension.

Demographic data and clinical severity descriptors versus functional outcome were tested in a binary logistic regression model.

**Results:**

In all, 428 major trauma patients participated in the study. At 1 year, 50.8% of trauma patients included had a good recovery and 49.2% had some degree of disability. The median value of quality of life was 0.725.

At the multivariate analysis, variables showing significant impact on functional outcome were age (*p* = 0.052, OR 1.025), injury severity score (*p* = 0.001, OR 1.025), and Glasgow coma scale ≤ 8 (*p* = 0.001, OR 3.509)

The Spearman’s Rank correlation coefficient showed a strong correlation between the global level of function variables and quality of life at one year (Spearman’s Rho Correlation Coefficient 0.760 (*p* < 0.0001)).

**Conclusions:**

Increased age, increased injury severity score, and severe traumatic brain injury are predictors of long-term disability.

Most of these trauma patients show impairments that affect not only the level of functional state but also the quality of life. The degree of functional independence has the greatest positive impact on quality of life.

According to our results, after the recovery a prompt recognition of physical and psychological problems with systematic follow-up screening programs can help patients and doctors in defining specific therapeutic-rehabilitation pathways tailored to meet individual requirements.

## Background

Severe injuries are the main cause of death in the first four decades of life [1] and are a major cause of potential loss of years of life [2]. Severe injuries represent a considerable public health burden, with significant personal and societal costs. Major trauma patients experience a 20% mortality rate overall, and many survivors remain permanently disabled [3].

In Italy, the estimated cost of trauma care accounts for about 7% of the overall public healthcare costs, representing one of its major components [4].

Important improvements in trauma care and in particular in the rate of successful outcomes have been achieved with the introduction of integrated trauma systems in many countries worldwide [5, 6].

The recent Italian ministerial legislation [7] points out that the organization of trauma networks according to the hub-and-spoke approach is the preferred model. According to the model, the concentration of patients in a few Level I trauma centers (TC) aimed at ensuring prompt and specialized care should improve patient outcomes [5, 6]

In 2002, the regional health service of Emilia Romagna (Italy) designed three trauma systems, headed by three Level I TCs, based on geographic location, previous organizational history, and presence of clinical expertise. Each of these organizations is referred to as a “Sistema Integrato Assistenza Traumi” (SIAT; Integrated System for Trauma Patient Care), representing a separate, specific Trauma System [8].

Involving the collaboration of many professionals across different disciplines and areas, the trauma pathway governed by the Trauma System is extremely complex. As a consequence, the organization and the clinical governance of the trauma network are pivotal points in the achievement of successful trauma care. In order to monitor the quality of trauma care in the Trauma System, outcomes assessment is essential. Quality indicators can be conceptualized as the description of specific clinical processes or outcomes of care that, when they occur, represent desirable events or unfavorable deviations from an established norm. Quality indicators on outcome can be expressed as quality of life, functional outcome, post-traumatic stress, and others [9]. Quality indicators may be perceived as “sentinel” events in patient care (such as the delay in performance of key tests or treatments, or unexpected deaths), which may be associated with poor outcomes and/or sub-optimal care [9]. Nevertheless, a structured and long-term follow-up of trauma patients aiming at the assessment of outcome is not a frequent practice.

In fact, over the last 10 years, most of the studies and registries have focused solely on survival rate and on the occurrence of primary outcomes during hospitalization [10, 11].

Therefore, the trauma follow-up (TFU) system was created within the Romagna SIAT starting from the year 2006 in order to monitor the trauma network and assess its long-term outcomes.

The aim of this paper is to evaluate the existence of correlations between epidemiological data, injury severity, and clinical assessment characterizing the acute phase and the long-term outcomes in trauma patients and secondly to explore the association between outcome variables.

## Methods

After approval from the Research Ethics Committee, we conducted a cross-sectional study over a 10-year period, including patients with severe trauma who survived and were discharged from the Level I Trauma Center Intensive Care Unit (ICU) in Cesena, Italy. We evaluated long-term outcomes, explored the existence of correlations between factors characterizing the acute phase and the long-term outcome, and explored the association between outcome variables.

**Fig. 1:**
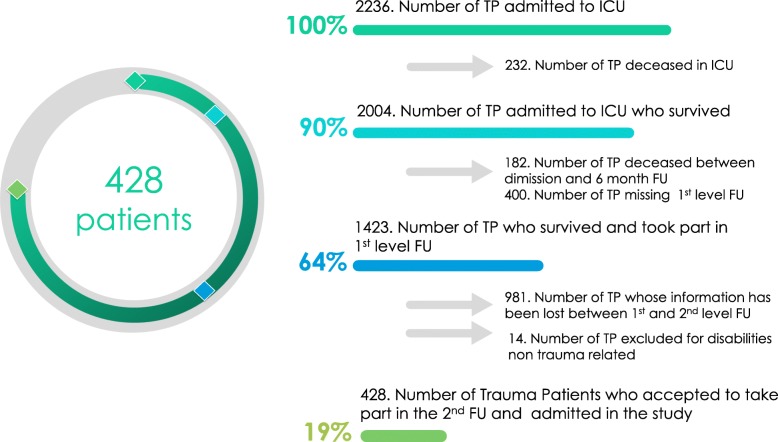
Number of trauma patients admitted to ICU, admitted to ICU who survived, who survived and took part in 1st level FU, and who accepted to take part in the 2nd FU and admitted in the study

The inclusion criteria were (1) traumatic injury with an Injury Severity Score (ISS) > 15, (2) admission to the level I TC ICU, and (3) trauma cases who followed the whole pathway of long-run outcome measurement at 1 year follow-up. The exclusion criterion was non-trauma-related disability. A total of 2236 trauma patients between January 2006 and December 2016 were admitted in Cesena ICU with an ISS >1 5; 232 patients died during the ICU stay, 182 patients died after ICU discharge, 442 patients concluded the entire follow-up, 14 had exclusion criteria, and 428 were analyzed (Fig. 1).

The detailed description of epidemiological data, severity of injury, and clinical variables characterizing the acute phase of the patients are shown in Table 1.

**Table 1:** Epidemiological data, severity of injury and clinical variables characterizing the acute phase

Trauma patients 2nd level FU			
Gender	*N* (%)	Male	318 (74.3)
		Female	110 (25.7)
Age	Mean (s.d.)		39.1 (20.1)
	Median (IQR)		26.5 (33)
Mechanism of injury	*N* (%)	Closed	422(98.6)
		Penetrating	6 (1.4)
Glasgow Coma Scale	Median (IQR)		9 (8)
Injury Severity Score	Median (IQR)		27 (14)
Multiple injury 2 body regions	AIS ≥ 3, *N* (%)		220 (51.4)
Hypoxia (SpO2 < 92%)	*N* (%)		224 (52.9)
Hypotension (SBP < 90 mmHg)	*N* (%)		127 (30.0)

*FU* follow-up, *N* number, *s.d.* standard deviation, *IQR* interquartile range

Demographic data and data concerning severity of trauma were collected from the patient data management system (PDMS) and from the Regional Trauma Registry.

The outcome measures were assessed with the use of the following:

- The Extended Glasgow Outcome Scale (GOS-E) is a global outcome scale assessing functional independence, work capabilities, social and leisure activities, and personal relationships. Its eight outcome categories rank as follows: GOS-E 1, death; GOS-E 2, vegetative state (unable to obey commands); GOS-E 3, lower severe disability (dependent on others for care); GOS-E 4, upper severe disability (independent at home); GOS-E 5, lower moderate disability (independent at home and outside the home but with some physical or mental disability); GOS-E 6, upper moderate disability (independent at home and outside the home but with some physical or mental disability, with less disruption than lower moderate disability); GOS-E 7, lower good recovery (able to resume normal activities with some injury-related problems); and GOS-E 8, upper good recovery (no problems) [12].
- The Euro Quality of Life scale 5 dimension (EQ-5D) is a standardized instrument for the measurement of generic health status and is designed for self-completion (the patient reports him/herself outcome measures). It has two main components: health care description and evaluation. Health status is measured in terms of five dimensions: mobility, self-care, usual activities, pain/discomfort, and anxiety/depression. In the evaluation section, the respondents evaluate their overall health status using the visual analog scale [13].
- The problem checklist (PCL) which is a self-reported score reflecting the impact of the impairment in the affective, cognitive, and physical spheres [14].

EQ-5D and PCL were reported by the relatives for the patients in the GOS-E class 2 (vegetative state).

### Statistical analysis

Statistical analysis was performed using the software IBM SPSS 22.0.

Data are reported as mean and standard deviation (SD), median, and interquartile range (IQR), number and percentage (*N*, %), depending on the underlying distribution. Patients’ clinical severity was described by the ISS, the coexistence of multiple injuries, the presence of hypoxia or hypotension in the early phase after trauma, and the Glasgow Coma Scale (GCS). Glasgow Outcome Scale extended outcome scale was dichotomized for data analysis (GOS-E class 2 and 3 = unfavorable outcome; GOS-E class 4, 5, 6, 7, and 8 = favorable outcome), according to Hutchinson et al. [15].

Since the first aim of the study was to understand what variables have an impact on long-term outcomes, demographic data and clinical severity descriptors were tested in univariated analysis versus GOS-E dichotomized.

Independent Student’s *t* test, Mann Whitney *U* test , and *χ*^2^ tests were used for statistical analysis.

Variables reporting a *p* value < 0.05 were tested in a binary logistic regression model.

Outcome variables were GOS-E dichotomized; a stepwise backward LR method was adopted, with significance value for exclusion < 0.1; age and ISS were tested as continuous variables according to Di Bartolomeo et al., 16 severe traumatic brain injury (defined as Glasgow Coma Scale ≤8), hypoxia, and hypotension as categorical variables.

Secondly, to test any correlation between the personal perception of outcome (EQ-5D) and functional outcomes (GOS-E), we measured Spearman’s rank correlation coefficient.

### Ethics approval and consent to participate

The FU system protocol was approved by the hospital administration.

All the procedures performed in the study were in accordance with the ethical standards of the institutional and/or national research committee and with the 1964 Helsinki Declaration and its later amendments or comparable ethical standards.

The study was submitted to the local Ethics Committee (CEROM, IRSST, Meldola, Italy- n.2480 del 24.07.2019 prot FU SYSTEM di AUSL Romagna), in accordance with its own indications. The study was observational and retrospective and was conducted on data collected according to the indications of the Italian regulatory board (http://www.garanteprivacy.it/web/guest). The data were made fully anonymous and de-identified before analysis.

Relatives accepted and signed our ICUs’ policy regarding data collection and follow-up interviews.

## Results

### Sample characteristics

A total of 428 patients were included in this study.

Frequencies and median values of the demographic, severity of injury, and clinical assessment for the full sample (*n* = 428) who completed the 2nd level FU included in the analysis are presented in Table 1.

### Descriptive statistics for multi-dimensional clinical outcomes

Of the 428 subjects with a 2nd level FU, 10 patients (2.3%) were coded as in a vegetative state (GOS-E = 2), 61 (14.2%) as severe disability lower (GOS-E = 3), 42 (9.8%) as severe disability upper (GOS-E = 4); moderate disability (GOS-E = 5 or 6) and good recovery ( GOS-E = 7 or 8) was 97 (22.7%) and 218 (51%), respectively (Table 2).

**Table 2:** Long-term outcome assessment

Trauma patients 2nd level FU			
Glasgow outcome scale extended	*N* (%)	GOS-E2	10 (2.3)
		GOS-E3	61 (14.2)
		GOS-E4	42 (9.8)
		GOS-E5	35 (8.2)
		GOS-E6	62 (14.5)
		GOS-E7	82 (19.2)
		GOS-E8	136 (31.8)
Affective PCL	Median (IQR)		17.5 (26.3)
	Prevalence (%)		86.6
Cognitive PCL	Median (IQR)		20 (36.6)
	Prevalence (%)		86.7
Physical PCL	Median (IQR)		19 (23.8)
	Prevalence (%)		91.7
EQ-5D	Median (IQR)		0.725 (0.674)

*FU* follow-up, *N* number, *IQR* interquartile range

The median value of quality of life (EQ-5D) was 0.725 (IQR 0.674).

The median PCL score of the affective, cognitive, and physical impairment was 17.5 (IQR 26.3), 20 (IQR 36.6), and 19 (IQR 23.8), respectively. The prevalence of patients who reported at least one checklist problem in each sphere, affective, cognitive, and physical, was 86%, 86%, and 91%, respectively. All results are listed in Table 2.

### Correlations between acute injury and clinical outcome variable

Results of univariate analysis between demographic data and clinical severity of the two groups (unfavorable and favorable outcome) are shown in Table 3. Gender, multiple injury, and hypotension was not associated to long-term outcomes.

**Table 3:** Univariate analysis between factors of the acute phase and long-term outcome (GOS-E)

		Unfavorable	Favorable	*p*
Patients	*N* (%)	71 (16.6)	357 (83.4)	
Gender, male	*N* (%)	50 (70.4)	268 (75.1)	0.413
Age	Mean (s.d.)	43.8 (21.7)	38.2 (19.6)	0.048
	Median (IQR)	41 (42.7)	36 (32.2)	
Injury severity score	Median (IQR)	33 (13)	26 (14)	0.008
Multiple injury (2 body regions AIS ≥ 3)	*N* (%)	41 (57.8)	179 (50.2)	0.242
Glasgow coma scale	Median (IQR)	5 (7)	9 (7)	< 0.001
Glasgow coma scale ≤ 8	*N* (%)	153(44.9)	48 (70.6)	< 0.001
Hypoxia	*N* (%)	47 (67.1)	177 (50.1)	0.009
Hypotension	*N* (%)	28.9 (102)	35.7 (25)	0.256

Variables reaching statistical significance were age, ISS, GCS, and hypoxia.

At the multivariate analysis level, variables showing significant impact on functional outcome were age (CI 1.010–1.040), ISS (CI 1.000–1.051), and GCS ≤ 8 (CI 1.870–6.585) (Table 4).

**Table 4:** Logistic regression analysis of long-term outcome (GOS-E) and covariates in trauma patients.

2nd Level TFU	*P*	OR	CI
Age	0.052	1 .025	1.010–1.040
ISS	0.001	1.025	1.000–1.051
Severe TBI	0.001	3.509	1.870–6.585

*ISS* Injury Severity Score, *TBI* Traumatic Brain Injury, *OR* odds ratio, *CI* confidence interval

### Association between clinical outcome variables

**Fig. 2:**
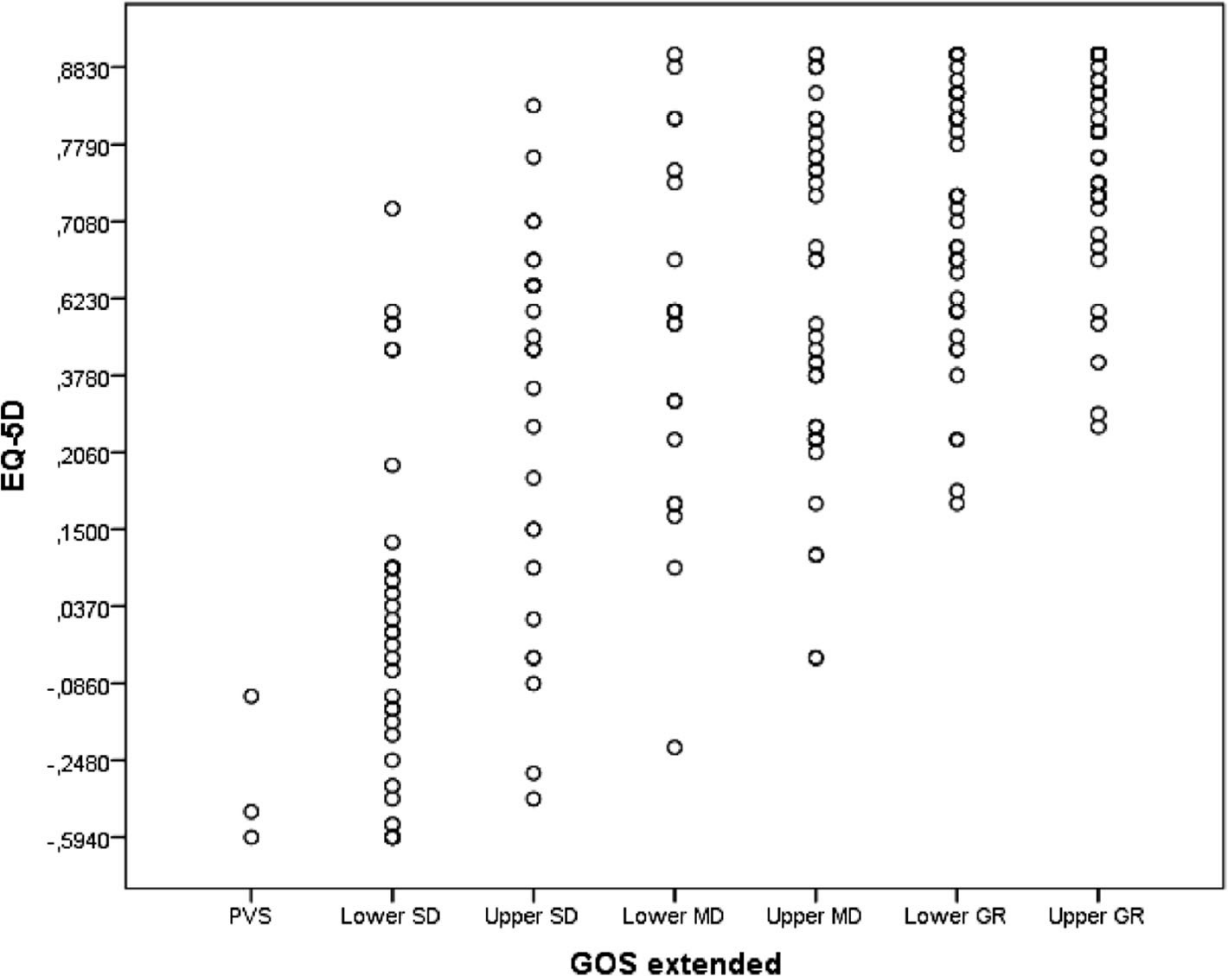
Correlations between the global level of function (GOS-E) and quality of life (EQ-5D) at 1 year

Correlations between the global level of function (GOS-E) and quality of life (EQ-5D) at 1 year are presented in Fig. 2. The diagram reports the scatter plot of the variable EQ-5D for different levels of GOS-E. The Spearman's Rank correlation coefficient showed a strong correlation between the two long-term outcome variables (Spearman’s Rho correlation coefficient 0.760 (*p* < 0.0001)).

## Discussion

Governing a trauma network is difficult and complex. In the Italian Healthcare Service, it is unusual to have data about long-term outcomes; the high cost and lack of a team of dedicated doctors and nurses make a structured 1- or 2-year follow-up study difficult to create.

Securely, injury has a long-term impact on functional state, return to a productive work, personal relationship, and social and leisure activities [16, 17]. In our study there is a high percentage (49%) of patients with some degree of disability and 34.5% do not return to their previous work. Most of these trauma patients show problems concerning their emotional, physical, and cognitive spheres that need to be carefully followed by trauma specialists within the trauma system.

These impairments affect not only the level of functional state by limiting the ability to perform activities, but also affect the patients’ quality of life [16]. The median EQ-5D index score in our study is 0.7 and there is a strong correlation between the quality of life and global level of functioning, confirming that the degree of functional independence has a great impact on quality of life. Prompt recognition of these problems with systematic follow-up screening programs can help in defining specific therapeutic-rehabilitation pathways.

Furthermore, in the acute phase these results can help health care providers to illustrate the illness trajectory to individual patients and their families as they identify their own goals of care and match the treatment provided to these goals [18].

The literature about long-term outcomes in trauma patients is sparse and is limited by the lack of an inclusive classification system for measuring disability or health outcomes [19], making a comparison of our results with other case studies difficult. Additional larger, multicenter studies are necessary to produce more robust correlations between trauma severity and treatment effect on long-term outcomes.

Advanced age, increased ISS score, and GCS ≤ 8 are positive predictors of long-term disability in our population of patients; in particular, GCS is the clinical variable that has the greatest impact on unfavorable outcomes. The initial neurological assessment therefore has an important prognostic value, suggesting the hypothesis that the greatest impact on the long-term outcome in survivors is determined by the severity of the head injury. A study with a wider range of samples and brain images data would be useful so as to better examine this hypothesis.

Although hypoxia and hypotension in the pre-hospital setting and/or at hospital admission are two main factors related to short-term outcomes, in particular mortality [20, 21], they do not prove to influence long-term outcomes in our population. However, the study is limited by the retrospectively gathered data on patients’ vital variables in the pre-hospital and emergency setting where operators are engaged in performing therapeutic maneuvers/procedures. This limitation could influence the accuracy of clinical data collected.

This study is also somehow limited due to the following facts: there is a low percentage of patients’ response to long-term follow-up, which points out the difficulty in including patients in studies with long-term assessments; secondly, the analyzed sample consists of strictly intensive care patients. We have been unable to assess the follow-up of all the patients admitted to Level 1 Trauma Centers due to the lack of data at the time of the study regarding hospitalized patients not in intensive care units.

Considering these findings, we are now proposing a better traceability system of the variables identified in this paper that influence the trauma outcomes. A “real time and hands-free” tracking system capable of recognizing the events and quantifying them temporally will be useful [22].

The road to functional recovery is complex. A first step, as proposed in this paper, is a comprehensive application of bio-psychosocial view of care to understand what the patients judge as a good outcome.

Future studies that examine patients’ perspectives on “good” outcomes would also contribute to health care providers’ ability to match treatment with the patient’s objectives.

## Conclusion

This study shows that age, ISS, and initial GCS are important determinants of the long-term trauma care outcome. In particular, the greatest impact on the long-term outcome in survivors is determined by the severity of head injury. Although hypoxia and hypotension in the pre-hospital setting and/or at hospital admission are two main factors related to short-term outcome as mortality [16, 17], they do not influence long-term outcome.

Most of trauma patients show problems concerning their emotional, physical and cognitive sphere that need to be carefully followed by trauma specialists within the trauma system. These impairments affect not only the functional state by limiting the ability to perform daily activities but also the patients’ quality of life. The degree of functional independence has a great positive impact on quality of life. The road to functional recovery is complex and requires a comprehensive application of bio-psychosocial view of care. What clinicians should consider is how patients judge their condition; a good outcome for patients differs from patients to patients. The heterogeneity of patients’ good outcome perception is a vital aspect that clinicians have to take into consideration: this point highlights the importance of the follow-up. According to our results after the recovery, a prompt recognition of physical and psychological problems with systematic follow-up screening programs can help patients and doctors in defining specific therapeutic-rehabilitation pathways tailored to meet individual requirements.

## Data Availability

The datasets used and/or analyzed during the current study are available from the corresponding author on reasonable request.

## Abbreviations

AIS: Abbreviated Injury Scale
EQ-5D: Euro Quality of Life scale 5 dimension
GCS: Glasgow Coma Scale
GOS-E: Glasgow Outcome Scale
ICU: intensive care units
ISS: Injury Severity Score
PCL: Problem checklist
PDMS: Patient data management system
SIAT: Integrated System for Trauma Patient Care (Sistema Integrato Assistenza Traumi)
TC: Trauma centers
TFU: Trauma follow-up
